# The Mutation Hotspots at UGT1A Locus May Be Associated with Gilbert’s Syndrome Affecting the Taiwanese Population

**DOI:** 10.3390/ijms232012709

**Published:** 2022-10-21

**Authors:** Paul Wei-Che Hsu, Po-Cheng Liao, Yu-Hsiang Kao, Xin-Yu Lin, Rong-Nan Chien, Chau-Ting Yeh, Chi-Chun Lai, Yu-Chiau Shyu, Chih-Lang Lin

**Affiliations:** 1 Institute of Molecular and Genomic Medicine, National Health Research Institute, Zhunan 350, Taiwan; 2 Community Medicine Research Center, Chang Gung Memorial Hospital, Keelung Branch, Keelung 204, Taiwan; 3 Liver Research Center, Chang Gung Memorial Hospital, Taoyuan 333, Taiwan; 4 College of Medicine, Chang Gung University, Taoyuan 333, Taiwan; 5 Department of Ophthalmology, Chang Gung Memorial Hospital, Keelung 204, Taiwan; 6 Department of Nursing, Chang Gung University of Science and Technology, Taoyuan 259, Taiwan; 7 Liver Research Unit, Keelung Chang Gung Memorial Hospital, Keelung 204, Taiwan

**Keywords:** Gilbert’s syndrome, genetic factors, single nucleotide polymorphism, machine learning

## Abstract

Gilbert’s syndrome is mainly diagnosed through genetic analysis and is primarily detected through a mutation in the promoter region of the *UGT1A1* gene. However, most of the research has been conducted on Caucasian populations. In this study, we studied the Han population in Taiwan to investigate the possibility of other mutations that could cause Gilbert’s syndrome. This study comprised a test group of 45 Taiwanese individuals with Gilbert’s syndrome and 180 healthy Taiwanese individuals as a control group. We extracted DNA from the blood samples and then used Axiom Genome-Wide TWB 2.0 array plates for genotyping. Out of 302,771 single nucleotide polymorphisms (SNPs) from 225 subjects, we detected 57 SNPs with the most significant shift in allele frequency; 27 SNPs among them were located in the UGT1A region. Most of the detected SNPs highly correlated with each other and are located near the first exon of *UGT1A1, UGT1A3, UGT1A6*, and *UGT1A7*. We used these SNPs as an input for the machine learning algorithms and developed prediction models. Our study reveals a good association between the 27 SNPs detected and Gilbert’s syndrome. Hence, this study provides a reference for diagnosing Gilbert’s syndrome in the Taiwanese population in the future.

## 1. Introduction

Gilbert’s syndrome (GS) is a hereditary disease. The most common physiological symptom is jaundice, caused by the toxic unconjugated bilirubin in the blood (hyperbilirubinemia). However, it does not cause any other abnormal liver functions [1]. Additionally, hepatic processing of drugs metabolized through glucuronidation may be affected in patients with GS, including acetaminophen, nonsteroidal inflammatory drugs, statins, gemfibrozil, human immunodeficiency virus protease inhibitors (indinavir and atazanavir), sorafenib, and irinotecan (CPT-11). It has been found that unconjugated bilirubin has a protective effect against cardiovascular disease, diabetes, and metabolic syndrome [2]. This can be primarily attributed to its antioxidant action and anti-inflammatory properties. However, the risk of GS associated with the neoplastic disease is controversial in several tumors [2]. Bilirubin is a waste product of heme catabolism [3]. The breakdown of hemoglobin releases heme, which is transformed into unconjugated (indirect) bilirubin. The unconjugated bilirubin is generally bound to glucuronic acid, in the liver, by the enzyme uridine diphosphoglucuronate glucuronosyltransferase (UGT) [4]. This enzyme converts the toxic form of bilirubin (unconjugated bilirubin) to its nontoxic form (conjugated bilirubin) and is encoded by the *UGT1A1* gene [5]. Conjugated (direct) bilirubin, which is water-soluble, passes from the liver to the gallbladder, where it is mixed with other constituents of bile and finally enters the small intestine [5,6]. Only a small fraction of it is transported into the kidneys and excreted with urine [7]. This contributes to the formation of tea or cola-colored urine and brown-colored stool [8]. However, if the *UGT1A1* gene mutates, it causes GS [9,10,11]. Changes in the promoter region of the *UGT1A1* gene lead to the dysfunction of the UGT enzyme [12,13]. When UGT enzyme dysfunctions, unconjugated bilirubin is not transformed into conjugated bilirubin. High levels of unconjugated bilirubin in the blood lead to jaundice, characterized by yellow discoloration of the skin and jaundice [14]. Caucasian and African American populations with GS are usually characterized by the homozygous form of the UGT1A1*28 allele (rs34983651), which is a homozygous 2-bp insertion (genotype (TA)7TAA/(TA)7TAA) mutation of the TATA box promoter region of the *UGT1A1* gene, resulting in higher concentrations of serum bilirubin [9,12,13,15,16,17]. Recent studies have shown that GS may be associated with *UGT1A7* as well [18]. To examine the genotypes of GS in the population of Taiwan, we used the Axiom Genome-Wide TWB2.0 array based on the genotyping data of the Taiwanese subjects [19].

## 2. Results

### 2.1. Clinical Characteristics

A total of 225 subjects were recruited for this study from August 2013 to April 2021. There were 45 patients with GS and 180 healthy controls. The patients’ GS diagnoses are based on clinical characteristics, with more details provided in Study Subjects. The level of total bilirubin in patients with GS was 1.5–2.8 mg/dL, and the ratio of direct bilirubin to total bilirubin ranged between 0.06–0.19. In comparison, in the healthy controls, the level of total bilirubin was 0.2–0.8 mg/dL, and the ratio of direct bilirubin to total bilirubin ranged between 0.5–1. The demographic and clinical baseline characteristics of the subjects are presented in Table 1. As we know, no significant differences were observed for the other clinical variables.

### 2.2. Genetic Variants Associated with GS

**Figure 1:**
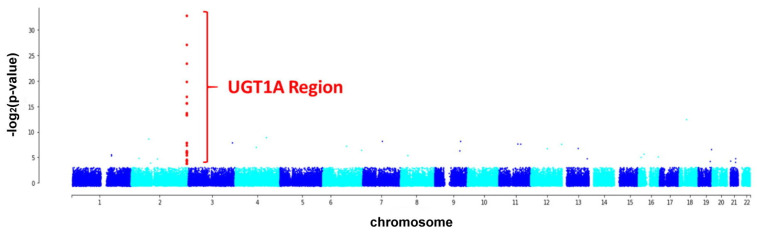
Manhattan plot visualizing the *p*-values of the allele frequency changes among the subjects with Gilbert’s syndrome (test group) and healthy individuals (control group). Most of the significant single nucleotide polymorphisms (SNPs) are located in the UGT1A region (chr2:233617645–233773305).

By analyzing the whole-genome SNPs, we found 57 SNPs whose allele frequency variations differed significantly between the test and control groups (*p* < 0.05, Table S1). Moreover, 27 out of the 57 SNPs were located in the UGT1A gene, and the difference was highly significant (Figure 1).

**Figure 2:**
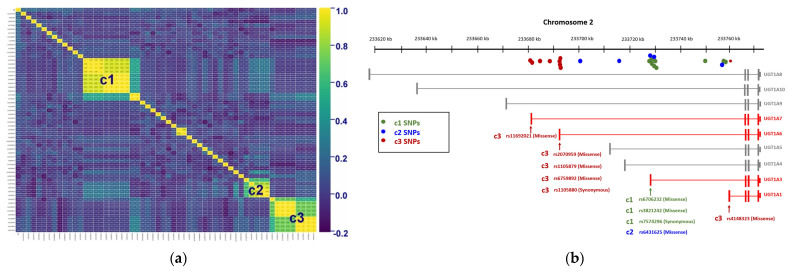
(**a**) Correlation matrix of 57 significant SNPs (*p* < 0.05). There are three clusters (c1, c2, and c3) of SNPs that are highly correlated with each other (*r* > 0.6). All clusters are located in the UGT1A region. (**b**) The graph shows the distribution of SNPs in the UGT1A region. The three clusters in the correlation matrix are represented by green (c1), blue (c2), and red (c3). Most c1 and c2 SNPs are clustered near the first exon of *UGT1A3*, and three of them belong to missense variants. Except for rs4148323, which is the missense variant of *UGT1A1*, the other c3 SNPs are concentrated between the first exon of *UGT1A7* and the first exon of *UGT1A6*, and most of them are missense variants of *UGT1A7* or *UGT1A6*.

We used the correlation matrix to represent the correlation between these 57 significant SNPs and found that three clusters (c1, c2, and c3) had a high correlation with each other (Figure 2a). The average correlation coefficient of c1 SNPs (n = 9) was 0.791, c2 SNPs (n = 5) was 0.786, and c3 SNPs (n = 9) was 0.804. These three clusters contained SNPs in the same location, the UGT1A region of the genome (Table 2). The UGT1A region includes nine genes: *UGT1A1*, *UGT1A3*, *UGT1A4*, *UGT1A5*, *UGT1A6*, *UGT1A7*, *UGT1A9*, *UGT1A10*, and *UGT1A8* (in descending order of gene length). Figure 2b shows that the SNPs in the UGT1A region gather around the first exon of the genes *UGT1A1, UGT1A3, UGT1A6*, and *UGT1A7*. Hence, the SNPs located near the 5′ end of these genes might be the cause of GS.

### 2.3. Machine Learning

**Figure 3:**
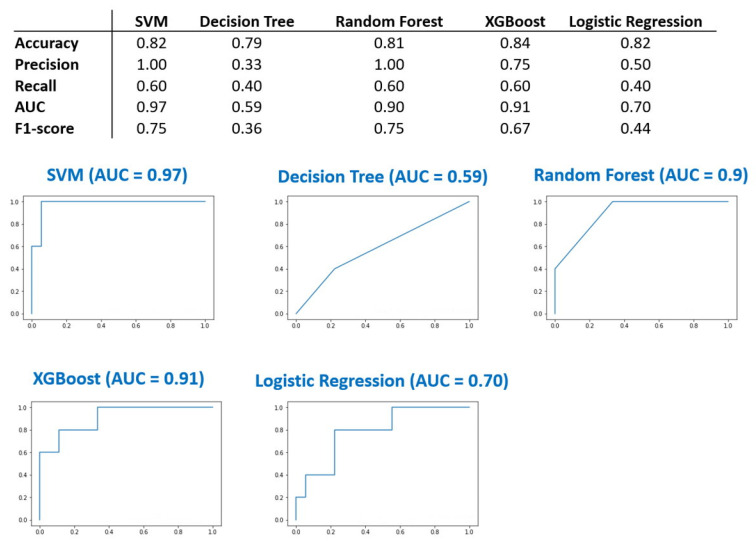
27 SNP signatures were used to build predictive models via five different machine learning methods. Support Vector Machine (SVM), Random Forest, and XGBoost showed better performance of accuracy and area under the receiver operating characteristic (ROC) curve (AUC).

The above results showed that the 27 SNPs located in the UGT1A region exhibit significant differences in the test subjects with GS and are highly correlated with each other. We used these 27 SNPs as input variables for the machine learning models. The prediction models for GS were developed using the algorithms SVM, Random Forest, Logistic Regression, Decision Tree, and XGBoost. SVM and XGBoost perform better in terms of accuracy rate and AUC (Figure 3). SVM and XGBoost displayed an accuracy rate of 0.82 and 0.84, respectively. The AUC of the SVM and XGBoost models were 0.9 and 0.91, respectively. The data demonstrate that these 27 SNPs are a reliable marker in the diagnosis of GS.

### 2.4. Limitations

Our study has several limitations. First, only a few subjects were available for analysis. Gilbert’s syndrome is a rare disease and a hereditary genetic disorder. In order to collect more information from the limited number of probes in the SNP array and small sample size, we hope to use whole genome sequencing (WGS) to obtain more SNPs information in the future. Second, validation cohorts of other races or places of residence may be warranted for evaluation in the future. Finally, the current study could not assess clinical events such as drug interactions, cardiovascular disease, diabetes, metabolic syndrome, and cancer outcomes. Therefore, we could not determine a GS-related effect in the above-mentioned clinical events. Moreover, Gilbert syndrome usually is diagnosed until puberty or later. We will design our research in the direction of cohort and longitudinal study so that we may also develop models to predict the opportunity to have Gilbert’s syndrome from childhood.

## 3. Discussion

We have identified novel biomarkers for the diagnosis of GS through this study. For genetic diagnosis, UGT1A1*28 mutations have been established as a diagnostic marker for GS [15,20]. The genotype (TA)7/(TA)7 insertion in the SNP rs34983651 in the promoter region of *UGT1A1* gene (chr2:233760233) promotes the conversion of (TA)6TAA into (TA)7TAA, which impacts *UGT1A1* activity and causes the mutations [12].

However, for the Taiwanese Nationwide Cohort examined in this study, we found that UGT1A1*28 or rs34983651 might not be the leading cause of GS. The genome-wide association study shows that although the mutations occur in the UGT1A region, the 27 SNPs identified in this study are positioned around the first exon of the genes *UGT1A1*, *UGT1A3*, *UGT1A6*, and *UGT1A7*. Studies have reported that mutations in the *UGT1A1* region are frequently associated with mutations in *UGT1A6*, *UGT1A3*, and *UGT1A7* [21,22,23]. This study has identified mutant hotspots in UGTs with greater precision. Out of the 27 SNPs, 8 (rs6706232, rs3821242, rs6431625, rs4148323, rs11692021, rs2070959, rs1105879, and rs6759892) belong to missense variants (Table 2), implying that these 8 SNPs directly affect the activity of these proteins. We used the allele frequencies of these 27 SNPs as the input for the machine learning algorithms to develop predictive models, and the resultant AUC was more than 90%. Since these SNPs have not been reported to be related to GS, we have developed reference data for the diagnosis of GS in the Taiwanese population.

## 4. Materials and Methods

### 4.1. Study Subjects

From August 2013 to April 2021, 225 prospective participants were recruited from the Northeastern Taiwan Community Medicine Research Cohort (NTCMRC, ClinicalTrials.gov Identifier: NCT04839796). They were then enrolled and examined in the gastroenterology clinic of the Community Medicine Research Center of Chang Gung Memorial Hospital, Keelung Branch. The participants were divided into 2 groups. The test group comprised 45 subjects with GS and the control group with 180 healthy subjects without GS, and male to female ratio (approx. 1.6:1) was the same in both groups. All subjects underwent clinical examination, blood tests, and assessment of complete individual medical histories. GS was ascertained at total bilirubin >1.4 mg/dL, the ratio of direct bilirubin to total bilirubin was <0.2, and significant jaundice symptoms were also observed, but the levels of glutamic oxaloacetic transaminase (GOT) and glutamic pyruvic transaminase (GPT) were in the normal range for the liver function tests. In addition, we did not find any abnormalities of the liver, gallbladder, pancreas, spleen, or kidneys or past medical records of chronic hepatitis. Informed consent was provided by all participants. This study conformed to the ethical guidelines of the 1975 Declaration of Helsinki and was approved by the Institutional Review Board of the Chang Gung Medical Foundation (IRB No: 201800802B0 and 202000077B0A3).

### 4.2. Clinical Assessment

Personal medical history and questionnaires were collected from 225 participants during recruitment. In addition, all of them were subjected to physical examination and biochemical tests for preliminary clinical evaluation.

### 4.3. DNA Extraction from White Blood Cells

Blood samples were collected in citrate-treated tubes and centrifuged at 3000 rpm for 10 min at 4 °C to separate the serum from the cells. Next, the erythrocytes were lysed prior to the phenol/chloroform-based (Sigma Aldrich, St. Louis, MO, USA) DNA extraction process. Lastly, we used 95% isopropanol (J.T. Baker Inc., Philadelphia, PA, USA), followed by 80% ethanol (J.T. Baker Inc., Philadelphia, PA, USA), to obtain the total genomic DNA.

### 4.4. Whole-Genome Single Nucleotide Polymorphism (SNP) Analysis

We used the Axiom Genome-Wide TWB 2.0 array plates (Thermo Fisher Scientific Inc., Waltham, MA, USA) for the genotyping of genomic DNA samples from the 225 subjects, which is the only means of identifying SNPs in the Taiwanese population, containing 681,796 SNPs. The SNPs whose minor allele frequency was zero and those with more than 10% missing rate were removed. The remaining 302,771 SNPs out of the total 681,796 SNPs were further analyzed.

### 4.5. Correlation Heatmap and Machine Learning

The genotype was converted to numerals based on the SNP data. The reference for homozygous was considered at 1, for heterozygous at 1.5, and for homozygous alternate at 2. Then, we used the programming language Python 3.8 with package scikit-learn 1.0.2 to analyze the converted data and plotted a correlation heatmap. In addition, we used the scikit-learn 1.0.2 package to develop five different machine-learning models using Support Vector Machine (SVM), Random Forest, Logistic Regression, Decision Tree, and XGBoost and calculated their accuracy, precision, recall, area under the receiver operating characteristic (ROC) curve (AUC) and F1-score to evaluate the performance of the models.

## 5. Conclusions

Our study reveals that the 27 SNPs play an important role in the Taiwanese population with Gilbert’s syndrome, which differs from the currently known UGT1A1*28 allele (rs34983651) as the basis of diagnosis for Gilbert’s syndrome in other populations. Hence, this study provides a reference for diagnosing Gilbert’s syndrome in the Taiwanese population in the future.

## Figures and Tables

**Table 1:** Baseline characteristics of 225 subjects.

	Healthy Control n = 180	Gilbert’s Syndrome n = 45	*p*-Value
Age, years	60.45 ± 14.90	67.49 ± 13.99	0.005
Female, n (%)	68 (37.8%)	17 (37.8%)	1
T_Bilirubin ^1^ (mg/dL)	0.63 ± 0.24	1.73 ± 0.30	<0.001
D_Bilirubin ^2^ (mg/dL)	0.22 ± 0.08	0.22 ± 0.06	0.719
D_Bilirubin/T_Bilirubin	37.82 ± 12.63	12.78 ± 3.08	<0.001
Anemia, n (%)	11 (6.1%)	2 (4.4%)	0.668
WBC ^3^ (×10^3^/μL)	6.11 ± 2.64	6.17 ± 1.95	0.885
RBC ^4^ (×10^3^/μL)	4.8 ± 0.60	4.86 ± 0.49	0.513
Hemoglobin (g/dL)	14.08 ± 1.42	14.3 ± 1.69	0.356
Hematocrit (%)	41.91 ± 3.69	42.16 ± 4.07	0.689
MCV ^5^ (fL)	88.04 ± 7.48	87.2 ± 8.14	0.504
MCH ^6^ (pg)	29.58 ± 2.91	29.57 ± 3.34	0.993
MCHC ^7^ (g/dl)	33.55 ± 1.03	33.86 ± 1.22	0.092
RDW ^8^ (%)	13.39 ± 1.03	13.58 ± 1.63	0.33
Platelets (×10^3^/μL)	244.28 ± 62.13	244.18 ± 62.05	0.992
Transferrin (ng/mL)	245.36 ± 34.75	235.14 ± 35.12	0.08

^1^ T_Bilirubin: total bilirubin; ^2^ D_Bilirubin: direct bilirubin; ^3^ WBC: white blood cells; ^4^ RBC: red blood cells; ^5^ MCV: mean corpuscular volume; ^6^ MCH: mean corpuscular hemoglobin; ^7^ MCHC: mean corpuscular hemoglobin concentration; ^8^ RDW: red blood cell distribution width.

**Table 2:** The information on SNPs among these tree clusterings which having a high correlation with each other.

Clustering	SNP	Chromosome	Position (bp)	*p*-Value	Location
c1	rs3755319	2	233,758,936	0.003031869	UGT1A region: Intronic Variant
	rs4124874	2	233,757,013	0.005113795	UGT1A region: Intronic Variant
	rs2221198	2	233,749,977	0.00333	UGT1A region: Intronic Variant
	rs7574296	2	233,729,603	0.002707358	UGT1A3: Synonymous Variant
	rs3806597	2	233,728,923	0.026915457	UGT1A region: Intronic Variant
	rs2008595	2	233,728,546	0.026915457	UGT1A region: Intronic Variant
	rs6706232	2	233,729,207	0.0152489	UGT1A3: Missense Variant
	rs3806596	2	233,729,061	0.015291563	UGT1A region: Intronic Variant
	rs3821242	2	233,729,157	0.012727593	UGT1A3: Missense Variant
c2	rs17863787	2	233,702,448	0.002450485	UGT1A region: Intronic Variant
	rs1976391	2	233,757,337	6.66 × 10^−15^	UGT1A region: Intronic Variant
	rs1875263	2	233,716,976	0.000759234	UGT1A region: Intronic Variant
	rs6431625	2	233,729,266	1.99 × 10^−12^	UGT1A3: Missense Variant
	rs1983023	2	233,728,376	8.00 × 10^−11^	UGT1A region: Intronic Variant
c3	rs4148323	2	233,760,498	0.00000151	UGT1A1: Missense Variant
	rs7586110	2	233,681,881	0.000000195	UGT1A region: Intronic Variant
	rs10168416	2	233,688,441	0.000000195	UGT1A region: Intronic Variant
	rs11692021	2	233,682,559	5.39 × 10^−8^	UGT1A7: Missense Variant
	rs2070959	2	233,693,545	0.000000195	UGT1A6: Missense Variant
	rs1105880	2	233,693,319	0.00000193	UGT1A6: Synonymous Variant
	rs1105879	2	233,693,556	0.00000193	UGT1A6: Missense Variant
	rs6759892	2	233,693,023	0.00000193	UGT1A6: Missense Variant
	rs4261716	2	233,684,471	0.00000133	UGT1A region: Intronic Variant

## Data Availability

Data available on request due to restrictions, e.g., privacy or ethics. The data presented in this study are available on request from the corresponding author. The data are not publicly available due to the Informed Consent Statement.

## Supplementary Materials

The following supporting information can be downloaded at: https://www.mdpi.com/article/10.3390/ijms232012709/s1.

## Clinical Trial Registration

Northeastern Taiwan Community Medicine Research Cohort (NTCMRC, ClinicalTrials.gov Identifier: NCT04839796).

## Abbreviations

GS	Gilbert’s syndrome
UGT	uridine diphosphoglucuronate glucuronosyltransferase
GOT	glutamic oxaloacetic transaminase
GPT	glutamic pyruvic transaminase
SNP	single nucleotide polymorphism
SVM	Support Vector Machine
ROC	receiver operating characteristic
AUC	area under the ROC curve.
