# Supplementary material for: The Mutation Hotspots at UGT1A Locus May Be Associated with Gilbert’s Syndrome Affecting the Taiwanese Population
Source: Int J Mol Sci. 2022 Oct 21;23(20):12709. doi: 10.3390/ijms232012709 (PMC9603874; doi:10.3390/ijms232012709)
Supplement: Supplementary file 1 [file ijms-23-12709-s001.zip › ijms-1913746-supplementary.pdf]

Table S1. 57 SNPs whose allele frequency variations have a significant difference between Gilbert's Syndrome and Healthy Control (p<0.05)

SNP	Chromosome	Position (bp)	p-value	Location
rs1976391	2	233757337	6.66E-15	UGT1A region : Non Coding Transcript Variant
rs6431625	2	233729266	1.99E-12	UGT1A3 : Missense Variant
rs1983023	2	233728376	8E-11	UGT1A region : Non Coding Transcript Variant
rs11692021	2	233682559	5.39E-08	UGT1A7 : Missense Variant
rs7586110	2	233681881	1.95E-07	UGT1A region : Non Coding Transcript Variant
rs10168416	2	233688441	1.95E-07	UGT1A region : Non Coding Transcript Variant
rs2070959	2	233693545	1.95E-07	UGT1A6 : Missense Variant
rs4261716	2	233684471	1.33E-06	UGT1A region : Non Coding Transcript Variant
rs4148323	2	233760498	1.51E-06	UGT1A1 : Missense Variant
rs1105880	2	233693319	1.93E-06	UGT1A region : Non Coding Transcript Variant
rs1105879	2	233693556	1.93E-06	UGT1A6 : Missense Variant
rs6759892	2	233693023	1.93E-06	UGT1A6 : Missense Variant
rs6506901	18	31233604	4.17E-06	LOC105372049 : Intron Variant
rs13103746	4	133198613	0.000143	PCDH10 : Intron Variant
rs6710960	2	74732928	0.00019	LOC102724497 : Intron Variant
rs10236324	7	82746808	0.000298	intergenic
rs12685739	9	110016181	0.000299	PALM2AKAP2 : Intron Variant
rs72549262	3	187235126	0.000398	MASP1: Intron Variant
rs2741047	2	233673008	0.000456	UGT1A region : Non Coding Transcript Variant
rs2741048	2	233673102	0.000474	UGT1A region : Non Coding Transcript Variant
rs10082670	11	80616789	0.000502	intergenic
rs4526739	11	92931854	0.000512	intergenic
rs77359010	12	131226370	0.000527	intergenic
rs1875263	2	233716976	0.000759	UGT1A region : Non Coding Transcript Variant
rs9390757	6	101540637	0.000774	GRIK2 : Intron Variant
rs10025824	4	89860387	0.000969	intergenic
rs7329085	13	68120560	0.001194	intergenic
rs2717417	12	70635651	0.001251	PTPRB : Intron Variant
rs7250003	19	56664116	0.001477	ZNF835 : Synonymous Variant
rs501296	6	165199622	0.001712	intergenic
rs3919995	9	106675687	0.001918	LINC01505 : Intron Variant
rs45625338	2	233729259	0.002294	UGT1A3 : Missense Variant
rs17863787	2	233702448	0.00245	UGT1A region : Non Coding Transcript Variant
rs7574296	2	233729603	0.002707	UGT1A3 : Synonymous Variant
rs3755319	2	233758936	0.003032	UGT1A region : Non Coding Transcript Variant

rs2221198	2	233749977	0.00333	UGT1A region : Non Coding Transcript Variant
rs2560407	16	24090861	0.003647	PRKCB : Intron Variant
rs2790054	1	165813205	0.004117	intergenic
rs145442045	8	31399504	0.004784	LOC101929492 : Intron Variant
rs2790057	1	165817074	0.005094	intergenic
rs4124874	2	233757013	0.005114	UGT1A region : Non Coding Transcript Variant
rs247898	16	84441603	0.006235	ATP2C2 : Intron Variant
rs34498969	16	11519017	0.006864	LOC400499 : Intron Variant
rs2168395	2	33514230	0.008341	RASGRP3 : Intron Variant
rs2850056	21	35960022	0.008868	LOC101928269 : Intron Variant
rs7337910	13	106460650	0.008969	intergenic
rs62160698	2	111433700	0.009475	MIR4435-2HG : Intron Variant
rs3821242	2	233729157	0.012728	UGT1A3 : Missense Variant
rs9982976	21	15062902	0.01443	NRIP1 : Intron Variant
rs6706232	2	233729207	0.015249	UGT1A3 : Missense Variant
rs3806596	2	233729061	0.015292	UGT1A region : Non Coding Transcript Variant
rs12983058	19	51139527	0.015459	SIGLEC9 : 3 Prime UTR Variant
rs12627317	21	35385120	0.018206	LOC100506403 : Intron Variant
rs116955810	2	233702369	0.019163	UGT1A region : Non Coding Transcript Variant
rs10180984	2	82196724	0.02127	intergenic
rs3806597	2	233728923	0.026915	UGT1A region : Non Coding Transcript Variant
rs2008595	2	233728546	0.026915	UGT1A region : Non Coding Transcript Variant
